# Putting into Words the COVID-19 Lockdown Experience: Psychological Symptoms and the Referential Process

**DOI:** 10.3390/healthcare9091100

**Published:** 2021-08-25

**Authors:** Rachele Mariani, Silvia Monaco, Michela Di Trani

**Affiliations:** Department of Dynamic and Clinical Psychology, and Health Studies, Sapienza University of Rome, 00185 Rome, Italy; silvia.monaco@uniroma1.it (S.M.); michela.ditrani@uniroma1.it (M.D.T.)

**Keywords:** referential process, psychological symptoms, writings, COVID-19

## Abstract

The coronavirus pandemic is a unique collective event which has affected the physical and psychological health of all individuals. Restrictions imposed by governments to counteract this situation have represented risk factors for developing psychopathological symptoms. This study aims to explore the relationship between psychological symptoms and the referential process (RP). Forty-eight healthy participants (25 males, mean age = 39.3; SD = 16.6) completed a demographic questionnaire and the Symptom Checklist-90-Revised (SCL-90-R) through an online platform and wrote about their experience 3 weeks after the imposition of the lockdown. Different linguistic measures of the RP were applied to the narratives. The logical functions expressed through written narratives (The Italian Reflection Dictionary score, IREF) showed significant positive correlations with the SCL-90-R General Score Index (GSI) and different SCL-90-R subscales (depression, anxiety, obsessive-compulsiveness, interpersonal sensitivity, hostility, and paranoid ideation). On the contrary, the reorganization and reflection function related to emotional events (The Italian Weighted Reflection and Reorganization List score, IWRRL) showed significant negative correlations with the SCL-90-R’s GSI and different subscales (obsessive-compulsiveness, depression, anxiety). The results highlight the relationship between psychological symptoms and complex defense mechanisms based on the intellectualization of negative emotions and a positive strategy of reorganization based on emotional elaboration. These results suggest the importance of supporting collective elaborations of citizens in the context of the pandemic.

## 1. Introduction

The coronavirus disease 2019 (COVID-19) produced critical effects, creating a worldwide health emergency. As the pandemic evolved, Italy has been particularly affected, with widespread social and health consequences. Institutions highlighted the impact of COVID-19 and related consequences on the mental health of the public [1], encouraging professionals and researchers to investigate ways to effectively cope with the related emerging problems. This was not an easy task, as the unique situation created by COVID-19 prevented the direct use of past knowledge of the literature. Stress, anxiety, and depression now have a common substrate among millions of people, and there is general bewilderment regarding the topic [2].

Several articles about the mental health effects of the COVID-19 pandemic have been published, showing a disastrous scenario around the world. Regarding the situation in Italy, alarming conditions emerged for both the clinical and non-clinical populations. More specifically [3], depressive and anxious symptoms increased, and when compared to past studies, post-traumatic stress symptoms became common amongst people with positive cases nearby, a general consolidation of psychopathology emerged, and many changes in sleeping patterns and insomnia appeared [4]. Moreover, some studies have deepened particular elements of the lockdown condition, where the confinement is predictive of internalizing symptoms, mediated by emotional dysregulation levels [5]. The COVID-19 pandemic has had a global impact. It is not possible to limit the research to an individual view of this phenomenon, excluding the community aspect. Many researchers explored a new form of joint change, using one of the most accessible resources of this era—language. From the start of the pandemic, the language used has changed [6], and it has been an important predictor of the public’s mental health [7].

Given the speed with which various governments have responded to the pandemic situation by imposing social distancing rules, many researchers have found it useful to investigate the emotional impact using new methods of investigation that do not require a pre-post measurement, as is usually done. In fact, they concentrated on linguistic diversity during the epidemic, both in social media in the form of posts or tweets [8,9] and through collecting interviews or writing. In fact, linguistic research focused on an important part of this mental health crisis and studied how people faced this event in their life [10]. Research highlighted the variety of emotions provoked by the COVID-19 pandemic [11], particularly the negative emotions that are also found in dream narratives [12]. These data suggest the importance of such a collective traumatic experience in re-processing the event in a cognitive way as well as an emotional one. This new research paradigm is in line with a growing body of evidence linking language analysis to psychopathology. In fact, the employment of specific phrases or narrative styles has been demonstrated to be diagnostically relevant. According to these studies, various diagnostic aspects can be predicted through language, such as the use of the personal pronoun “I” for depression [13] or the use of abstract phrases for affective detachment in stressful conditions [14,15]. Several studies have found a strong link between language, psychopathology, and somatic disorders [16], thus showing that language may be used to evaluate psychological processes because it is an indicator of mind–body functioning [17].

Bucci developed an interesting theory for understanding the functioning of the mind, the Multiple Code Theory (MCT) [18], which was the starting point for later computerized linguistic measures of what is referred to as the referential process (RP). The MCT includes three systems for processing and representing emotional information: the nonverbal sub-symbolic (visceral sensations experienced during an arousal related to an emotion); the nonverbal symbolic (symbols, images); and the verbal symbolic (words). According to Bucci’s theory, RP is a process which interconnects these three processing systems. The RP’s final aim is to bring the information from a nonverbal form to an image and then transform it into language [19,20]. RP is not one-way, but works in a bidirectional way, so that the verbal material can be translated to a nonverbal form. The RP can be activated by interpersonal relationships and, also, by psychotherapy [21]. It can be defined by three principal phases: emotional arousal, narrating/symbolizing, and reflecting/reorganizing. In the MCT, it is hypothesized that trauma or conflicts can disturb an appropriate development of the three systems, which is also disruptive to the RP’s functioning. This kind of disconnection can be applied to both somatic and mental pathology [22,23,24], and it could be increased if there are aversive experiences for negative events and emotions, since some distancing processes are activated [25]. The greater the disconnection is, the more a person feels emotional arousal without being able to communicate their experience with words. The RP can be reactivated by interpersonal communication, proceeding from an emotion arousal phase to a symbolic one, in which the emotional experience is put into images and words, and finally passes through a phase of reorganization and reflection which expands the meanings attributed to the experience.

Research exists on the relationship between linguistic measures and psychopathology [26,27]. Bucci’s MCT focused the majority of its research on the evaluation of psychopathology throughout time and in the therapeutic context. However, the literature shows and encourages the consideration of the role of RA in the analysis of psychopathology [28,29,30]. A gap in the literature currently exists regarding the role of RA in the consolidation of psychopathological traits during the COVID-19 lockdown.

The general objective of this study was to examine the relationship between linguistic features and psychopathology during one of the most stressful and traumatic experiences, the COVID-19 pandemic. We analyzed whether the linguistic style, measured by computerized dictionaries of RP, would correlate to psychopathology symptoms. In particular:

1. Based on previous research, we expected to find a significant positive correlation between psychopathology and logical and reflective language, as a measure of the defense mechanisms of intellectualization and rationalization.
2. We expected to find a significant positive correlation between somatization, depressive symptoms, somatic language and negative effects, as measures of verbal expression of body parts, symptoms and negative feelings.

Moreover, considering these aims from an exploratory point of view, we also compared two subsamples with high and low symptomatology, respectively. This was done by using the median-split of the general score for the psychopathological traits, and the Global Severity Index (GSI) as a cut-off for splitting the sample on linguistic style dimensions, in order to identify specific linguistic features.

## 2. Materials and Methods

### 2.1. Procedure

The study was conducted in the first week of April 2020, 3 weeks after the onset of governmental lockdown restrictions. This choice in timing was motivated by the will to explore the effects of the lockdown experience, rather than the immediate reactions. It seemed appropriate to give some weeks for the elaboration and stabilization of the effects on the participants. The study was conducted online through Google Forms, where the participants provided the informed consent and accepted the privacy policy before beginning the task. Participants then completed an open-ended question before completing the self-report measures. The open-ended question, which did not limit the number of words typed, was, “Considering your current situation of the COVID-19 pandemic and lockdown rules, how are you experiencing this difficult time?” This phrase was designed to allow people to think and write about the traumatic condition more freely. Recruitment for this study stopped at the end of the lockdown (18 May 2020). Ethical approval was granted by the ethics committee of the University “Sapienza” of Rome, Department of Dynamic, Clinical and Health Psychology.

### 2.2. Participants

The sample was recruited from the general Italian population and participants were invited to complete an online form. The form was advertised as a study on health during COVID-19 and was distributed through a link, via snowball sampling. In order to respond to the form, participants had to confirm that they were: following the social distancing rules of the lockdown; between the ages of 18 and 70; living in Italy during the lockdown; had never had a psychiatric diagnosis or psychopharmacological treatment; and were not a healthcare professional during the pandemic. A total of 48 individuals participated in the study, 25 males and 23 females. All of their written responses were considered to be acceptable for data analysis, according to the adherence with the open-ended question. Participants had a mean age of 39.3 (SD = 16.6); the youngest was 18 years old and the oldest was 41 years old. Moreover, 52.08% of participants were married/cohabiting, 25.00% were unmarried, 18.75% were single, and 4.17% were divorced/separated.

### 2.3. Measures

#### 2.3.1. Sociodemographic Questionnaire

The self-administered questionnaire collected data from multiple variables, such as age, gender, and level of education.

#### 2.3.2. The Symptoms Checklist-90-Revised (SCL-90-R)

The Symptoms Checklist-90-Revised [31] is a self-report inventory composed of 90 items, rated by the subject on a 5-point Likert scale, ranging from 0 (not at all) to 4 (extremely). The SCL-90 usually takes about 15 min to complete. The checklist consists of symptoms reported in the past week, which are divided among nine dimensions: somatization (SOM), obsessive-compulsive (OC), interpersonal sensitivity (IS), depression (DEP), anxiety (ANX), hostility (HOS), phobic anxiety (PHO), paranoid ideation (PAR) and psychoticism (PSY). The sum of all 9 subscales creates the Global Severity Index (GSI; Cronbach’s alpha: 0.96), which reflects general psycho-somatic distress. Higher levels reflect increased psycho-somatic distress.

#### 2.3.3. Italian Discourse Attributes Analysis Program (IDAAP) and Linguistic Measures of the Referential Process

In order to empirically measure RP, several computerized measures have been developed in Italian (as well as in English) to characterize the phases of the RP. These include measures of symbolization, weighted dictionaries depicting reflection/reorganization language, and dictionaries depicting affect, reflection, disfluency, and sensory-somatic language. These dictionaries were developed and validated following a procedure in which lists of words were rated by judges according to operationalized descriptions of the constructs measured by each dictionary [32,33,34,35]. The IDAAP software allows the simultaneous application of more dictionaries and produces derived measures and the covariations between the two dictionaries. In our study, we used the following dictionaries and derived measures (see Table 1 for a summary) built and appropriately validated for the Italian language:

- The Italian Weighted Referential Activity Dictionary (I-WRAD) contains 9596 words and represents the computerized measure of RA. The scale ranges between 0 and 1, with 0.5 as the neutral value. The most frequent words depicting a low IWRAD score are associated with a subjective focus. Instead of pointing to external objects and describing situations, the focus is on the present rather than the past tense. High scores represent a high level of referential activity.
- The Italian Reflection Dictionary (IREF) is composed of abstract words concerning how people think and communicate their thoughts. This dictionary includes basic logic words and words referring to cognitive or logical functions or the failures of these functions.
- The Italian Sensory Somatic Dictionary (ISensD) is composed of words related to the body and bodily activities. The number of ISensD words in a speech sample measures the sub-symbolic activation.
- The Italian Sum Affect Dictionary (ISAffD) concerns how people feel and communicate their feelings. It includes words indicating an emotional response, either positive or negative. ISAffD is composed of three sub-dictionaries: positive affect (IAffP), negative affect (IAffN), and neutral affect without a specific valence (IAffZ).
- The Italian Weighted Reflection and Reorganization List (IWRRL) refers to the reorganization and reflection function, where the speaker is trying to recognize and understand the emotional value of an event or set of events. It is not an abstract reflection, but rather, an individual’s reasoning related to a vivid experience. High scores for this measure represent high reflection/reorganization.

### 2.4. Statistics

The statistical analyses were conducted using the Statistical Package for Social Science (SPSS), Version 26 for Windows (IBM, Armonk, NY, USA). Data were reported as frequencies and percentages for discrete variables and as means and standard deviations for continuous variables. A Pearson’s correlation was performed between SCL-90-R subscales, linguistic measures, and age. To address the levels of psychopathology, the sample was split in two groups (according to the median of GSI): the high group and the low group. This choice is in line with the nature of the sample (non-clinical) and previous literature [36,37] in order to explore a qualitative aspect of linguistic analysis related to higher and lower symptomatology. Then, an independent samples t-test was performed.

## 3. Results

### 3.1. Demographics and Descriptives

SCL-90-R levels appeared appropriate for a non-clinical population. The sample mean of SCL-90R was 0.61 (SD = 0.47) and the GSI percentile distribution was 25% (0.22); 50% (0.55); 75% (0.94). According to the clinical cut-off of SCL-90-R (GSI > 1), only eight participants (17%) showed significant levels of symptomatology. More specifically, more than 30% of participants displayed elevated symptoms of depression, anxiety, and obsessive compulsiveness. SCL90 did not show any significant differences when comparing the levels of education, gender assigned at birth, or relationship status.

### 3.2. Psychological Symptoms and Linguistic Measures

The application of linguistic measures to the narratives produced by participants during the lockdown experience brought interesting information to the forefront, regarding relationships between RP and psychopathological traits in this peculiar context. Pearson’s correlations between SCL-90-R and the RP measures are shown in Table 2. The abstract and reflection dictionary (IREF) correlated positively with the Global Severity Index (GSI), and with all the SCL-90-R subscales, except for phobic anxiety (PHO), psychoticism (PSY) and somatization (SOM). However, a weighted measure of the RP reorganization phase (IWRRL) correlated negatively with the Global Severity Index (GSI), and with the obsessive-compulsive (OC), depression (DEP), and anxiety (ANX) subscales. There were also no significant correlations with the affect and psychosomatic dictionaries. The correlations between sociodemographic and psychological variables produced only one significant correlation between age and positive affects (AffP), as shown in Table 2.

In order to analyze the linguistic measures in a normal sample, we conducted a median-split GSI (0.52) to define two groups, the low and high SCL-90 groups. The t-test for independent samples showed coherent results, as reported in Table 3: the group with high levels of SCL-90-R symptoms (high group) had significantly lower scores in referential activity (IWRAD) and reflection-reorganization (IWRRL) compared to the group with low symptomatology (low group). Consistent with these findings, the high group showed higher scores for the abstract-reflection dictionary (IREF) compared to the low group, which showed a higher use of abstract and cognitive words.

### 3.3. Narrative Vignettes

#### 3.3.1. Low Group

I have faith in the professionals who are dealing with the emergency; both doctors and the government. I am at home with my partner and living together is going well. I believe that if I had been stuck at home with my family, I would have suffered the situation more (I am out of the office). I am happy that at least one in four professors is proceeding with online video conferencing lessons because it allows me to have a routine and stimuli. I miss physical contact with people (friends, colleagues and family), but I am happy to be able to take advantage of technology to not feel completely isolated.

#### 3.3.2. High Group

My mood is affected every other day by the unusual situation. I live everyday life with relative serenity trying to occupy the time in the most fruitful way. Sometimes I find it difficult to sleep at night and I accept, with impatience, the impossibility of being able to carry out the activities that I normally perform in the open air and in company. I followed all the rules carefully and scrupulously from the first days of the emergency.

## 4. Discussion

The social distancing implemented in many countries during the COVID-19 pandemic had a considerable impact on the general mental health of citizens.

The text analysis showed relevant results connected to psychopathology, as supposed. The first hypothesis was to verify if the abstract-reflection dictionary (IREF) correlates with some psychopathological indices. In the literature, the IREF dictionary showed abstract and intellectualized emotional defense processes [38]. In fact, in the psychotherapeutic process, a higher index of the abstract-reflection dictionary (IREF) in the therapists’ notes pointed to a defense mechanism of the intellectualizing of the therapist regarding the psychotherapeutic relationship [39,40]. The same results have been found in expressive writings during the COVID-19 pandemic: this index is strictly related to people who have more infected friends and relatives [41]. Our results are consistent with the current literature, which states that the abstract-reflection dictionary (IREF) is a measure of intellectualizing psychological defense, therefore creating distance from a stressful and unpredictable situation. In fact, the IREF dictionary is more related to the Global Severity Index, thus characterizing the written style of the more highly symptomatic group. This element appears in the clinical vignette, in which more abstract language is used in an attempt to explain the event, thereby avoiding an emotional reevaluation of one’s own experience. Moreover, a hostile reaction is strongly correlated with the IREF: anger may be the first emotional reaction connected to the impotency of staying at home and being unable to do anything to solve the situation.

The second hypothesis, regarding the relationship between somatization, depressive symptoms, the sensory-somatic dictionary, and negative affects, is not supported by the results. The current literature suggests that higher senso-somatic and affective words were directly related towards depression in unipolar and bipolar groups [30]. In healthy individuals, even when in stressful situations, linguistic features did not emerge. Most likely, the initial reaction of healthy individuals is a more cognitive and reflective one and less of an overwhelming feeling. The absence of this result in this first survey could be linked to the fact that a non-clinical population is investigated in this study, for which psychopathological symptoms are not stable in people but reactive to the contingent situation.

The last hypothesis regarded the explorative relations between the linguistic analysis and psychopathology. People with the highest Global Severity Index (GSI) scoring showed specific linguistic patterns; lower referential activity (IWRAD) and reflection/reorganizing (IWRRL); and higher scores for the abstract-reflection dictionary (IREF). These results are consistent with the psychological literature which assesses the presence of symptoms in people with more defense mechanisms [42]. This study also supports more recent literature on COVID-19 and defense mechanisms [43], showing different ways in which the general population faces with this traumatic situation [44]. In the frame of MCT, lower symbolization and reflection/reorganization reflect a general moment where a disconnected pattern can start and a strong use of intellectualization, with cognitive and abstract words, can be the first step in avoiding the impact of the activation of stressful and overwhelming emotional schemas. The clinical vignettes indicate this process and the High Group example shows a more detached description of the situation, distancing their own experience using abstract and more intellectualized terms.

In conclusion, these results seem relevant to describe the initial impact of an unknown event, such as a pandemic, on health through linguistic features. Replicating the same study today would most likely bring different results to light. For instance, one year after the onset of the virus, the depressive and distressing dimension would probably emerge with greater force. At the time of their response, individuals were unaware that the pandemic situation would continue for so long and as intensely. At the time of the study, individuals were using the defense mechanism of rationalization as their protective function. Moreover, these results suggest the importance of implementing psychological interventions one year after the start of the pandemic to support the emotional processing of the pandemic event, and to allow for access to the subjective experience, a potentially very painful one, initially contained from the defensive reactions.

These results seem to suggest the need for future research to focus on the relationship between psychopathological symptoms and RA, especially considering the defense mechanisms emerging from the narratives.

## 5. Limitations

Given the small sample size, our findings have a qualitative and exploratory value that requires larger samples in order to be verified. Moreover, the bias of high self-selection in this procedure must be disclosed. As previously suggested, there are several limitations to this cross-sectional method study; a long-term follow-up (one year) on the psychological symptoms could represent a relevant step towards understanding the causal link between psychopathology and linguistic features. The last important limitation is related to the use of a single self-report tool, the SCL-90-R, for the evaluation of psychopathological symptoms, instead of a broader set of measures.

## 6. Conclusions

In sum, on the basis of these findings, we can argue that a linguistic analysis of narratives can be an interesting new field of investigation to develop linguistic markers which can help clinicians to listen and help people who are suffering. Although our data may only have qualitative value, they display an important relationship between language and the emotional processing of the traumatic experience. Our findings are consistent with those of Cohn et al. [45], who investigated the linguistic markers of the psychological change surrounding the 9/11 terroristic attack. The study by Cohn et al. [45] was completed through analyzing the diaries of 1084 U.S. users of an online journaling service. They found an increase in cognitive processes related to the upsetting event, such as psychological distancing using more abstract words. Listening to and exposing language as a diagnostic indicator certainly opens up new ways of assessing and monitoring clinical interventions.

## Figures and Tables

**Table 1:** Examples of words belonging to Italian linguistic measures of the referential process.

**IWRAD**
High weight: odore (smell); urla (scream); baciare (to kiss). Medium weight: il (the); capisci (you understand); io (I). Low weight: superficiali (superficial); ansiosa (anxious); carini (nice).
**IWRRL**
High weight: impedisce (prevent); spensierata (carefree); bisogni (needs). Medium weight: chiaramente (clearly); discorso (speech); logicamente (of course). Low weight: sapendo (understanding); nascosta (hidden); separazione (separation).
**IRefD**
Attenzione (attention), capire (to understand), decisione (decision), ragione (reason), razionalità (rationality), ricordo (memory).
**ISenSD**
Ammalato (sick), digerire (digest), disorientamento (disorientation), dolore (pain), ridere (to laugh), sintomi (symptoms), vomitare (to throw up).
**ISAffD:**
Positive—abbracci (hugs), affidabile (reliable), felice (happy); negative—abbandonato (abandoned), depresso (depressed), impaurito (frightened); neutral—attesa (expectation), bisogno (need), coinvolto (involved)

Note: IWRAD = Italian Weighted Referential Activity Dictionary; IWRRL = Italian Weighted Reflection and Reorganization List; IRefD = Italian Reflection Dictionary; IDFD = Italian Disfluency Dictionary; ISenSD = Italian Sensory-Somatic Dictionary; ISAffD = Italian Dictionary of the Sum of Positive, Negative, and Neutral Affects, respectively.

**Table 2:** Pearson’s correlations between SCL-90-R, age, and linguistic measures of RA (N = 48).

SCL-90-R ^1^	Linguistic Measures ^2^							
	IWRAD	IRef	IWRRL	IAffN	IAffP	IAffS	IAffZ	ISensS
SOM	−0.062	0.207	−0.043	0.151	−0.138	−0.092	−0.092	0.109
OC	−0.176	0.372 **	−0.343 *	0.226	−0.059	−0.012	−0.080	−0.055
IS	−0.138	0.309 *	−0.278	0.171	−0.084	−0.035	−0.125	−0.184
DEP	−0.207	0.339 *	−0.347 *	0.182	−0.099	−0.036	0.000	−0.053
ANX	−0.151	0.367 *	−0.338 *	0.265	−0.095	0.010	0.158	−0.050
HOS	−0.178	0.520 **	−0.193	0.126	−0.204	−0.158	0.009	−0.105
PHO	−0.104	0.133	−0.236	0.147	−0.009	0.054	0.146	−0.149
PAR	−0.065	0.367 *	−0.237	0.187	−0.093	−0.037	−0.104	−0.080
PSY	−0.125	0.281	−0.136	0.144	−0.072	−0.027	−0.065	−0.046
GSI	−0.170	0.358 *	−0.290 *	0.205	−0.116	−0.049	−0.051	−0.064
AGE	0.283	−0.010	0.208	−0.104	0.290 *	0.244	−0.075	−0.018

^1^ SOM = somatization; OC = obsessive-compulsive; IS = interpersonal sensitivity; DEP = depression; ANX = anxiety; HOS = hostility; PHOB = phobic anxiety; PAR = paranoid ideation; PSY = psychoticism; GSI = Global Severity Index. ^2^ IWRAD = Italian Weighted Referential Activity Dictionary; IWRRL = Italian Weighted Reflection and Reorganization List; IRefD = Italian Reflection Dictionary; IDFD = Italian Disfluency Dictionary; ISenSD = Italian Sensory-Somatic Dictionary; ISAffD = Italian Dictionary of the Sum of Positive, Negative, and Neutral Affects, respectively. * *p* < 0.05 ** *p* < 0.01 (two-tailed).

**Table 3:** Differences in linguistic measures among two groups of SCL-90-R scores, the high group and low group.

Linguistic Measures ^1^	High Group (N = 25)		Low Group (N = 23)		t	Sign
	M	SD	M	SD		
IWRAD	0.824	0.059	0.861	0.073	1.94	0.047 *
IRef	0.043	0.030	0.025	0.016	−2.62	0.012 *
IWRRL	0.730	0.089	0.793	0.078	2.59	0.013 *
IAffP	0.021	0.031	0.071	0.206	1.18	0.241
IAffN	0.044	0.056	0.029	0.042	−1.03	0.307
IAffS	0.073	0.069	0.102	0.201	0.67	0.505
IAffZ	0.008	0.015	0.002	0.006	−1.62	0.111
ISenS	0.065	0.041	0.057	0.044	−0.638	0.527

^1^ IWRAD = Italian Weighted Referential Activity Dictionary; IWRRL = Italian Weighted Reflection and Reorganization List; IRefD = Italian Reflection Dictionary; IDFD = Italian Disfluency Dictionary; ISenSD = Italian Sensory-Somatic Dictionary; ISAffD = Italian Dictionary of the Sum of Positive, Negative, and Neutral Affects, respectively. * *p* < 0.05; degrees of freedom = 46.

## Data Availability

The datasets used and analyzed during the current study are available from the corresponding author on request.
